# Assessment of Quality of Obturation, Instrumentation Time and Intensity of Pain with Pediatric Rotary File (Kedo-S) in Primary Anterior Teeth: A Randomized Controlled Clinical Trial

**DOI:** 10.5005/jp-journals-10005-1558

**Published:** 2018

**Authors:** Lavanya Govindaraju, Ganesh Jeevanandan, Subramanian EMG, Satish Vishawanathaiah

**Affiliations:** 1,3 Department of Pedodontics, Saveetha Dental College, Tamil Nadu, India; 2 Department of Pedodontics and Preventive Dentistry, Saveetha Dental College, Tamil Nadu, India; 4 Department of Pedodontics, Jazan University, Saudi, Kingdom of Saudi Arabia

**Keywords:** Children, Pain, Primary anteriors, Pulpectomy, Rotary file

## Abstract

**Aim:**

The purpose of this study was to clinically compare and evaluate the efficiency of pediatric rotary file kedo-S with hand K files and ProTaper rotary files during root canal preparation of primary anterior teeth.

**Materials and methods:**

Forty-five children requiring pulpectomy in any one of the vital primary anterior teeth were included. The children were randomly assigned to one of the three groups (15 per group), where the instrumentation was done using hand files in group 1, ProTaper rotary files in group 2 and Kedo-S rotary files in group 3. The instrumentation time, pain perception and the quality of obturation was assessed, and the statistical analysis was done using statistical package for social sciences (SPSS) software.

**Results:**

Statistically decreased instrumentation time was observed with the use of Kedo-S rotary file system (*p* < 0.05) when compared to the other two file systems. The intensity of pain experienced during instrumentation was also lesser with Kedo-S rotary file (*p* < 0.05). No significant difference was noted in the obturation quality with the exclusive pediatric rotary file when compared with the other two groups (*p* > 0.05).

**Conclusion:**

Kedo-S rotary files decrease the instrumentation time and intensity of the pain perception during canal instrumentation which positively influences the cooperation of the children.

**Clinical significance:**

Use of exclusive pediatric rotary files (Kedo-S) for root canal preparation in primary teeth reduces the fatigue of both the children and the dentists. Also, influences the behavior of the children in a more positive way thereby instilling a positive dental attitude.

**How to cite this article:**

Govindaraju L, Jeevanandan G, Subramanian EMG, Vishawanathaiah S. Assessment of Quality of Obturation, Instrumentation Time and Intensity of Pain with Pediatric Rotary File (Kedo-S) in Primary Anterior Teeth: A Randomized Controlled Clinical Trial. Int J Clin Pediatr Dent, 2018;11(6):462-467.

## INTRODUCTION

Early childhood caries (ECC) is a common oral health condition existing globally associated with several risk factors. The most commonly affected teeth are the maxillary primary anterior. Involvement of maxillary primary anterior is a classical manifestation of severe-ECC.^1^ The carious lesion in the primary anterior progresses rapidly and eventually leads to pathological fracture of the crown.^2^

Restoration of the anteriors serves as a challenge for the pediatric dentists owing to the age of the affected children. The ultimate goal of restoring the extensively carious primary anterior teeth rather than extracting is to preserve the teeth in its position until exfoliation, thus preserving the integrity of the dental arches. Hence, pulpectomy should be considered as the choice of treating decayed primary teeth despite being a strenuous and time- consuming procedure in pediatric dentistry.^3^

The success of the procedure highly depends on the cooperation of the child which is directly related to the length of the appointment.^4^ Conventionally the canal preparation of the primary teeth during pulpectomy was done using hand files and is the most time-consuming step of the procedure.^5^ The evolution of Ni-Ti rotary systems for canal preparation have resulted in rapid cleaning and shaping of the primary canals and is been widely accepted as an efficient tool in pediatric dentistry.^6^

*In vitro* and *in vivo* studies with rotary instrumentation for canal preparation in primary molars have shown reduced instrumentation time and more conical canal preparation that favors a better quality of obturation.^2,6–18^ However, all the above studies have been done using the rotary file designed for its use in permanent teeth.

An exclusive pediatric rotary with modified length, taper and tip size has been advocated for more conveniently and efficiently performing pulpectomy in primary teeth. With the constant progression in the endodontic field of child dentistry, an exclusive pediatric rotary system – Kedo-S (Reeganz Dental Care Pvt. Ltd., India) was launched. This system consists of three sets of files- D1, E1, and U1. The former was for the canal preparation of the primary molars, while the later was indicated exclusively to prepare the primary maxillary anterior.^19^

Only a handful of studies have been done evaluating the efficiency of rotary files for canal instrumentation in anterior.^5^ There are no clinical studies in the literature evaluating the efficiency of the use of Kedo-S rotary file in primary anterior teeth. Hence, the aim of the present study was to evaluate the instrumentation time, the quality of obturation, and also to record the pain experienced by the children during canal preparation of the primary maxillary anterior using Kedo-S exclusive pediatric rotary system.

## MATERIALS AND METHODS

This double blinded randomized clinical trial was conducted after obtaining approval from the Institutional Review Board (STP/SDMDS2015PED42) in the Department of Pedodontics and Preventive Dentistry in a dental institution. The patients’ parents were explained regarding the required treatment for their children and the participation of their children into the study was completely voluntary. The informed consent was obtained from the parents of the participated children before the start of the procedure.

### Patient Selection and Randomization

**Fig. 1:**
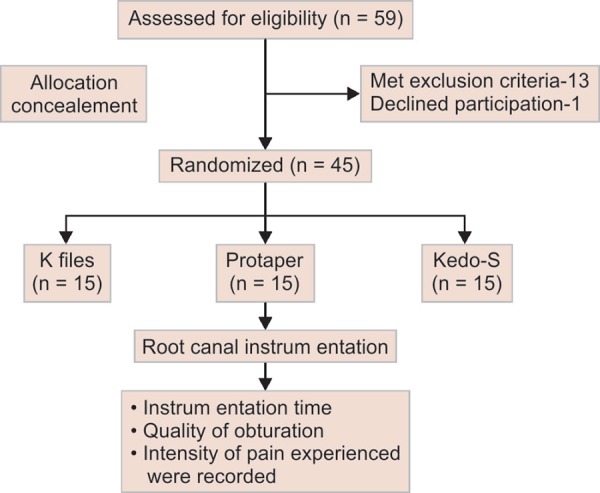
Consort flow diagram

A total of 45 children (15 per group) between the ages of 4–6 years with vital primary anteriors requiring pulp therapy were included in this study. Non-vital teeth, teeth with abscess, sinus tract, internal, and pathologic root resorption were excluded from the study. Also, children lacking the understanding ability, children with physical and psychological disabilities were excluded from the study. Computer generated randomization sequence was generated by a person, who was not a part of this study, and the teeth were randomly allotted to one of the three groups where the instrumentation was done using Hand files in group 1; ProTaper in groups 2 and 3 the instrumentation was carried out using Kedo-S Rotary system. A consort flow diagram is presented in Figure 1.

### Clinical Procedure

Root canal preparation for all the teeth was carried out by a single pediatric dentist in a single visit. Topical local anesthetic agent (Precaine B, Pascal International, USA) was applied, and the teeth were anesthetized with a local anesthetic solution containing 2% lignocaine with 1:200,000 adrenaline (LOX × 2% Adrenaline, Neon Laboratories Limited, India). The superficial caries was removed using high-speed sterile no. 330 round carbide burs (Mani Inc., Japan). The vitality of the tooth was confirmed by the presence of hemorrhage in the canal after de-roofing. No. 25 size K file (Mani Inc., Japan) was used to determine the patency of the canal. Working length determination was done with the help of preoperative radiograph and was kept 1 mm short of the apex.

In group 1, the canals were prepared using K-Hand files (Mani Inc., Japan) till size 50 in quarter pull turn method. The canals were irrigated with saline between each file size.

In group 2, single ProTaper rotary file system was used (Dentsply India Pvt. Ltd., Delhi, India). Only F1 ProTaper file was used for the preparation of the canal till the working length using an X-Smart motor (Dentsply India Pvt. Ltd., Delhi, India).

In group 3, the canal preparation was done using U1 Kedo-s rotary file (Reeganz Dental Care Pvt. Ltd.) till the working length using an X- Smart motor (Dentsply India Pvt. Ltd., Delhi, India).

The canals were then dried thoroughly using size 40 sterile paper points (Adenta). A mixture of calcium hydroxide and iodoform paste was used to obturate the root canals (Metapex, Meta Biomed Co., Ltd., Korea). A postoperative radiograph was taken to assess the quality of obturation. Glass Ionomer cement–type 9 (Shofu Inc. Kyoto, Japan) was used to seal the access cavity and a permanent restoration was done using appropriate strip crowns (3M ESPE Pediatric strip crowns).

### Assessment of Clinical Parameters

**Fig. 2:**
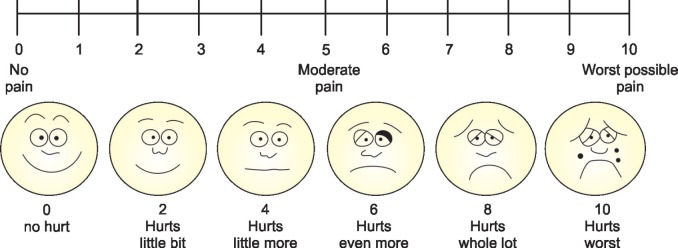
Consort flow diagram

The quality of obturation was assessed by a pediatric dentist who was blinded to the type of instrumentation used for canal preparation. The obturation quality was assessed as under-fill, over-fill or optimal-fill according to Coll and Sadrian criteria.^20^ During the canal preparation the instrumentation time was recorded in seconds using a stop watch by the assistant. The children and their parents were blinded to the type of instrumentation used. After the canal preparation, the child was assessed for the perception of pain during instrumentation using Wong Baker FACES Pain Scale. This self- reported scale was explained to the children and was asked to point out the face that depicts their pain level during the canal instrumentation (Fig. 2).

### Statistical Analysis

Kruskal–Wallis test was used to compare the instrumentation time and the pain experienced on instrumentation with different systems and the comparison of quality of obturation was done using the Chi-square test. Statistical analyses were performed using SPSS software version 17.0 (Chicago. SPSS Inc.). The statistical significance was set at *p* < 0.05.

## RESULTS

This study included a total of 22 boys and 23 girls with the mean age of 4.51 ± 0.727 years and the distribution of the teeth between the three groups is also tabulated in Table 1.

**Graph 1:**
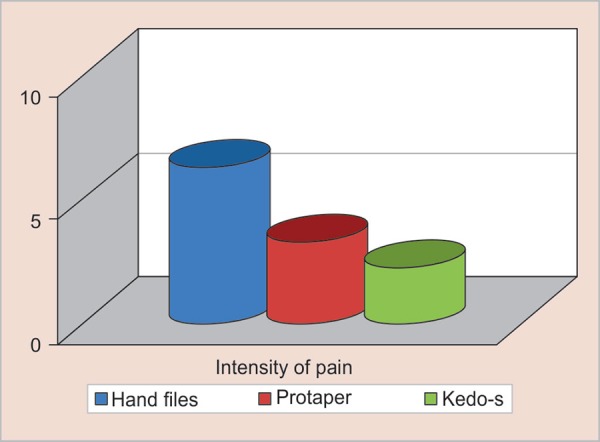
Intensity of pain experienced during canal instrumentation among different groups wherein Kedo- S file group resulted in less pain intensity compared to other groups

The mean instrumentation time on using a different file system for canal preparation was recorded. A statistically significant difference is noted between the three groups with regard to the instrumentation time (*p* < 0.05) as shown in Table 2. On evaluating the pain experienced by the children during canal preparation, it was found that with the use of Kedo-S rotary file the pain perception was statistically lower when compared to the other two systems (*p* < 0.05) as shown in Graph 1. However, no statistically significant difference was observed in the quality of obturation on using hand files, ProTaper, and Kedo-S rotary file for canal preparation in primary teeth (*p* > 0.05) as described in Table 3.

**Table 1:** Demographic and clinical features of the patients receiving different root canal preparation techniques

	*Hand file*	*ProTaper*	*Kedo-S*	*p value*
	*Age*			
*(Mean + SD)*	4.33 + 0.724	4.13 + 0.352	5.07 + 0.704	< 0.001*
	*Gender*			
*Male*	15	5	2	< 0.001 *
*Female*	0	10	13	
*Teeth*	*Hand file*	*ProTaper*	*Kedo-S*	*p value*
51	5	3	3	0.684 *
52	3	4	4	
53	0	0	2	
61	5	4	2	
62	2	3	3	
63	0	1	1	

^*^p < 0.05, statistically significant; Kruskal–Wallis test

**Table 2:** Comparison of instrumentation time among three groups

*Instrumentation time (in seconds)*	*N*	*Mean*	*Standard deviation*	p value*
Hand File	15	41.93	10.491	< 0.001* (Sig.)
ProTaper	15	19.60	9.387	
Keso-S	15	14.80	4.144	

*p < 0.05, statistically significant; Kruskal–Wallis test

**Table 3:** Comparison of quality of obturation among the different root canal preparation techniques

*Quality of obturation*	*Hand Files*	*ProTaper*	*Kedo-S*	*Total*	*p value*
Under-fill	40% (n = 6)	53.3% (n = 8)	26.7% (n = 4)	18	0.569*
Optimal-fill	33.3% (n = 5)	20% (n = 3)	46.7% (n = 7)	15	
Over-fill	26.7% (n = 4)	26.7% (n = 4)	26.7% (n=4)	12	

*p > 0.05, statistically not significant; Chi-square test

## DISCUSSION

The major apprehension of the parents in pediatric patients is the loss of primary anterior teeth affecting the esthetics and function, and also causes psychological depression for the child and the parent. It becomes the prime duty for a pediatric dentist to preserve the decayed primary anterior teeth. Pulpectomy is considered to be the treatment of choice for a primary tooth with pulpal involvement. The success rates for pulpectomies in primary anterior teeth were reported to be 77.7% and 86.2% by Flaitz et al.^21^ These findings support the fact that primary anterior pulpectomy is a viable alternative for extraction.

Root canal treatment in primary teeth is both challenging as well as time-consuming in children, eminently during the canal preparation. Over the years, an array of techniques and instruments were used for canal preparation of the primary teeth. Application of Ni-Ti rotary file systems for canal preparation in primary anterior was advocated by Bahrololoomi et al.^5^ It was also proved that the canal preparation in anterior teeth with rotary files was similar to that of manual files and the mean instrumentation time reduced with rotary files.*5,21* However, the rotary files used in the literature till date is designed for permanent teeth. A newly designed rotary file with alteration in the length and taper was advocated for its use in the primary teeth.^19^ Hence in this study, an exclusive pediatric rotary file designed for its use only in primary anterior teeth is compared with the Hand files and ProTaper files, designed for the permanent tooth. Instrumentation time, quality of obturation, and the pain experienced during instrumentation were evaluated in the study wherein, hand files and ProTaper files served as the control groups as these two systems are more conventionally used by the pediatric dentists.

Treatment time is an important aspect of practicing child dentistry. Reduced chair side time increases the cooperativeness of the children for dental treatment. Hence, assessment of the canal instrumentation time was done to find out the most reliable system that can be used in the primary teeth. In the current study, the mean instrumentation time was found to be significantly less in the group instrumented with Kedo-S rotary file. This reduction in the instrumentation time will create a positive impact on the behavior of the children. The instrumentation time with Kedo-S rotary file is much lesser than the ProTaper rotary file. This can be ascribed to the reduced working length (flute length) of the exclusive pediatric file, which is about 12 mm.^19^ A decrease in the length of the Kedo-S file allows easy insertion and removal of the file into the oral cavity of the children, making the treatment much easier and simpler for both the dentists as well as the children.

Endodontic operative discomfort is the degree of pain experienced by the patients during an endodontic procedure. In children, the intensity of pain experienced defines their behavior, which in turn determines the success of the treatment. The existing studies in the literature describe the techniques and outcomes of instrumentation with various files in primary anterior teeth but have not reported on the instrumentation pain intensity.*5,21* Hence, the pain on instrumentation was assessed in the current study to substantiate the use of various files for canal preparation in primary anterior teeth. In the present study, it was illustrated that there was a decrease in the pain perception by the children when instrumented with Kedo-S rotary file. The intensity of the pain experienced by the children was lesser with Kedo-S group due to the altered length and taper of the exclusive pediatric rotary file. U1 file, which is designed exclusively for the canal preparation of the primary upper anterior teeth has a variably variable taper with a tip diameter of 0.40 mm and is of 16 mm in length.^19^ These alterations in the rotary file make the canal preparation step less uncomfortable for the children resulting in lesser pain. This decreased pain experienced by the children will again create a positive impact on their behavior and also instill a positive dental attitude.

Lastly, the outcome of the procedure done is yet another important factor that has to be considered to arrive at conclusion. No significant difference was noted in the quality of obturation between the three groups. This could be attributed to the cleaning capacity of the different file systems. It is already stated in the literature that there is no difference in the cleaning capacity of the hand and rotary files.*5,21* Hence, these results do not negatively influence its use in primary teeth. The quality of obturation can be better assessed using three-dimensional imaging, which is a potential limitation of the present study. Also, in this study, there was an unequal distribution of the participants statistically with respect to the age and gender between the three groups which could have confounded the results of the present study.

## CONCLUSION

Based on the results of the present study, the following conclusions are drawn:

- Canal preparation with Kedo-S rotary file results in decreased instrumentation time and also reduces the intensity of the pain experienced by the children on instrumentation which in turn significantly improves the co-operation of the children towards dental treatment.
- The quality of obturation of the primary anterior canals with Kedo-S exclusive pediatric rotary file is similar to that of Hand files and Protaper rotary system indicating that the cleaning efficiency of Kedo-S rotary file is similar to the existing file systems.
- Further research evaluating the various other aspects Kedo-S rotary file has to be conducted to adjudge its use in the children.
